# Magnetic Field Intervention Enhances Cellular Migration Rates in Biological Scaffolds

**DOI:** 10.3390/bioengineering11010009

**Published:** 2023-12-22

**Authors:** Amy M. Vecheck, Cameron M. McNamee, Renee Reijo Pera, Robert J. Usselman

**Affiliations:** 1 Department of Biomedical Engineering and Sciences, Florida Institute of Technology, Melbourne, FL 32901, USA; 2 Department of Mathematics, California Institute of Technology, Pasadena, CA 91125, USA; 3 McLaughlin Research Institute, Great Falls, MT 59405, USA; 4 Department of Chemistry and Chemical Engineering, Florida Institute of Technology, Melbourne, FL 32901, USA; 5 Computational Research At Florida Tech (CRAFT), Florida Institute of Technology, Melbourne, FL 32901, USA

**Keywords:** cellular migration, radical pair mechanism, quantum biology, image analysis, computer vision, 3D bioscaffolds, magnetic mitohormesis

## Abstract

The impact of magnetic fields on cellular function is diverse but can be described at least in part by the radical pair mechanism (RPM), where magnetic field intervention alters reactive oxygen species (ROS) populations and downstream cellular signaling. Here, cellular migration within three-dimensional scaffolds was monitored in an applied oscillating 1.4 MHz radiofrequency (RF) magnetic field with an amplitude of 10 µT and a static 50 µT magnetic field. Given that cellular bioenergetics can be altered based on applied RF magnetic fields, this study focused on a magnetic field configuration that increased cellular respiration. Results suggest that RF accelerated cell clustering and elongation after 1 day, with increased levels of clustering and cellular linkage after 7 days. Cell distribution analysis within the scaffolds revealed that the clustering rate during the first day was increased nearly five times in the RF environment. Electron microscopy provided additional topological information and verified the development of fibrous networks, with a cell-derived matrix (CDM) visualized after 7 days in samples maintained in RF. This work demonstrates time-dependent cellular migration that may be influenced by quantum biology (QB) processes and downstream oxidative signaling, enhancing cellular migration behavior.

## 1. Introduction

The study of interactions between living systems and magnetic fields has a long history, culminating in a more advanced understanding of fundamental quantum processes in cell metabolism [1,2]. Magnetic field intervention drives the interplay between calcium signaling, reactive oxygen species (ROS), and mitochondria bioenergetics, resulting in magnetic mitohormetic responses [3,4]. At the core of the paradigm, some metabolic processes utilize the radical pair mechanism (RPM) to activate molecular oxygen using reduced flavin enzymes and produce ROS [5,6,7]. Hence, spin-selective ROS signaling channels are responsible for altering biological systems at the functional, cellular, and organism levels [2,8]. Recent evidence has further supported the RPM and ROS hypothesis in cells [9,10,11], circadian clocks [12], organisms [13,14], and mouse models [15], exemplifying a novel domain of quantum biology (QB). Therefore, QB is poised for integration into many applications, such as biotechnology [16], technobiology [17], and biomedical engineering [4], including regenerative medicine [18]. This work presents a novel method to investigate the RPM and cellular migration using quantitative image analysis in a tissue model of fibroblast cells embedded within a natural polymer scaffold. 

The RPM has been used to rationalize organism magnetosensitivity [19,20] and avian navigation [21,22], with cryptochrome flavoprotein as a potential magnetic sensor [23,24]. Briefly, the RPM occurs between radical pair (RP) intermediates that can be initialized by either photo-activation or during light-independent redox cycles [9,25,26]. A key feature of the RPM is the coherent transition between singlet and triplet states, with the ability to modulate singlet–triplet mixing with internal and external magnetic fields. RP spin dynamics can be impacted by weak static magnetic fields and oscillating magnetic fields, affecting downstream biological chemical processes. For ROS production within the RPM, singlet channels produce hydrogen peroxide (H_2_O_2_), whereas the triplet channels produce superoxide (O_2_^•−^) products. Singlet–triplet modulation occurs when resonant radiofrequency (RF) waves are applied to the RP on Zeeman or hyperfine resonance. However, biological RP reactions must satisfy specific physical and chemical requirements to accomplish magnetic sensing and with sufficient coherence times, presumably > 1 µs [27,28,29]. Therefore, biological responses can be sensitive to RF resonance with appropriate experimental magnetic field parameters, where the point of intervention occurs from quantum coherent RPs initializing from normal biological redox processes [30]. 

In previous work, we measured H_2_O_2_ and O_2_^•−^ levels with hyperfine resonance frequencies (7.0 MHz) and the resulting impact on cellular proliferation [1]. Our subsequent study demonstrated an increase in mitochondrial respiration by parallel fields at Zeeman resonance (1.4 MHz), whereas increases in glycolysis were observed by perpendicular fields [2]. Cellular bioenergetics was determined based on changes in the oxygen consumption rate (mitochondrial respiration) and extracellular acidification rate (glycolysis). Both studies verified a relative partitioning of increased H_2_O_2_ and decreased O_2_^•−^ levels, a hallmark of triplet-born spin-correlated ROS RPs on resonance and in the absence of visible light, i.e., dark reactions [26]. Static and oscillating magnetic fields will have different partitioning of ROS products that depend on the type of magnetic experiment and RP initialization [9,24,27,31]. Analogous ROS investigations have shown the impact of pulsed magnetic fields on mitochondria function [3,12]. Overall, evidence is accumulating that supports the RPM’s involvement in altering ROS levels and signaling channels in redox cell biology [3,7,9,12,14,15,18,23,24,31,32,33,34]. 

**Figure 1:**
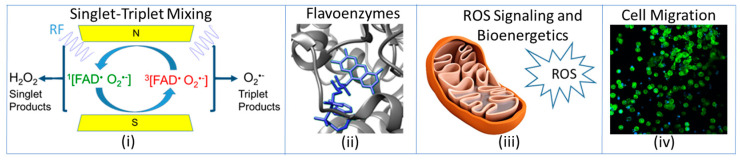
QB illustration of cellular spatial–temporal domains that link the RPM, ROS, and QBC cellular timing in living systems. (**i**) RPM singlet–triplet mixing is altered by resonant frequencies and redistributes ROS products for oxidative signaling. (**ii**) The activation of molecular oxygen by reduced flavins is the site of ROS generation. (**iii**) Oxidative signaling readjusts mitochondria bioenergetics between glycolysis and respiration. (**iv**) Downstream magnetic field intervention can accelerate cellular migration.

A new paradigm, called ROS QB, is emerging that connects the RPM at the quantum level to the classical spatial-temporal domains in living systems. Magnetic field control of ROS oxidative signaling on cellular bioenergetics offers novel opportunities to study many of the downstream signaling effects, such as cellular migration. In Figure 1, ROS QB is visualized as follows: (i) RF induces spin-mediated ROS partitioning via the RPM, (ii) occurring at flavoenzymes that satisfy spin physics constraints [35,36], (iii) ROS signaling acts analogously to a rheostat that regulates bioenergetics between respiration and glycolysis, and (iv) increased respiration and ATP production impacts cytoskeleton activity to accelerate cellular migration. By transitioning across the quantum/classical interface, the quantum signatures are written as classical outcomes occurring at the biomolecular, mitochondrial, and cellular levels. As the quantum events (ROS partitioning) percolate through the spatial domains (ROS signaling), the temporal domains manifest the magnetic field intervention at different cellular times. Therefore, the entire ROS QB paradigm can be cast into a novel type of cellular clock, as seen below. 

Although recent evidence from other laboratories [7,9,14,15,16,33] supports our ROS QB hypothesis, many fundamental unknowns remain. Guided by our previous results in ROS QB, we present a novel platform with regard to biological timing and clocks. Traditional biological clocks are commonly termed molecular clocks (digital and most often protein- or epigenetic-based) or time clocks (analog or circadian-based). With the emergence of our work in QB and the integration of key concepts of molecular and time clocks, we developed a framework for what we call a quantum biological clock (QBC). Here, the QBC will serve as the foundation for the molecular and time clocks, in terms of their interrelated function in cell metabolism. There exists much evidence for a QBC; however, the impact of quantum phenomena on biological functions has not been rigorously investigated. We propose that stimuli, distinct from genetically coded information, significantly impact cellular function. These analog signals may include redox statuses in oxidative signaling [30], ion concentrations [37], magnetic spins [7], and local electric fields [38]. This emerging paradigm suggests that non-genetic mechanisms influence the time dependence of information transfer [39]. For example, impairing the cellular NAD^+^/NADH redox balance slows the molecular clock by impairing protein synthesis and delays embryonic development [40]. In this way, the time required to transfer information may be equally or more important than the coded message itself. The hypothesis is to apply ROS QB principles to change the QBC timing, demonstrating one use for regenerative bioengineering.

To further understand the role of the RPM and QBC in cellular function, we used low-field and radiofrequency (RF) magnetic resonance to monitor cellular cluster rates within a fibroblast 3D bioscaffold. Fibroblasts are primarily responsible for extracellular matrix (ECM) remodeling, which is a pivotal design attribute in tissue engineering [41]. We hypothesize that increasing mitochondrial respiration will increase ATP production and, therefore, impact cytoskeleton function and cellular migration rates. Computer vision algorithms were used to determine cell distance distributions to calculate the migration rate, providing a quantitative measurement of collective cellular movement. Cells exposed to these specific magnetic field conditions resulted in accelerated scaffold degradation by increasing cell-derived matrix (CDM) production from clustered cells. Increased CDM formation was visualized as a mesh-like material over and around scaffold pores. This work demonstrates novel methodologies to study ROS QB processes and the impact of downstream effects of magnetic mitohormesis and the QBC in cellular migration. 

## 2. Materials and Methods

### 2.1. Cell Culture

NIH/3T3 mouse fibroblasts (ATCC) were seeded at a density of 6600/cm^2^ per T-75 flask containing 10 mL of Dulbecco’s Modified Eagle Medium (DMEM) supplemented with 10% fetal bovine serum (FBS) and 1% penicillin-streptavidin at 37 °C and 5% CO_2_. At approximately 80% confluency, the cells were passaged and seeded in alginate–gelatin hydrogels. 

### 2.2. Bioink Synthesis

Alginate–gelatin hydrogels were synthesized at 5% (weight/volume) for the alginate and gelatin composition and mixed at a ratio of 2:3, respectively [42]. Briefly, 0.2 g sodium alginate (Acros Organics, Thermo Fisher Scientific, Waltham, MA, USA) was added gradually to the solution of 10 mL PBS buffer and heated to 40 °C. A total of 0.3 g of gelatin (Sigma-Aldrich, St. Louis, MO, USA, gelatin from porcine skin) was added to the alginate solution and gently stirred until dissolved. In a 3 mL syringe, 1 mL of the alginate–gelatin mixture was aspirated, and the syringe was attached to a cell mixer kit (Cellink, Gothenburg, Sweden). In a second 3 mL syringe, 200 μL of cell culture media at a cell density of 2 × 10^6^ cells/mL was aspirated, and the syringe was attached to the opposite end of the stopcock. Plunges were pressed in an alternating pattern to form a homogenous solution mixture. The bioink solution was maintained in one syringe for the immediate extrusion process.

### 2.3. Bioprinting-Based Construct Formation

Constructs were formed using an extrusion-based methodology to distribute bioink in a layer-by-layer fabrication method, which does not alter the mechanical properties of the construct [43]. Using the 3 mL syringe filled during the bioink synthesis stage, 1 mL of bioink was extruded in a 15 mm × 15 mm × 4 mm 3D-printed (Creality Ender, Shenzhen, China) polylactic acid mold. Extrusion was completed in 10 s to minimize shear stress on the cells. Full crosslinking was conducted by submerging the print in 0.5 M CaCl_2_ for 10 min. All floating constructs were maintained in dual incubators containing either a static 50 μT magnetic field control or a static 50 μT and 1.4 MHz (10 µT amplitude) magnetic field until further analysis. 

Cellular live/dead assays (Thermo Fisher Scientific, Waltham, MA, USA) were tested on days 0, 1, and 7 to determine viability. Briefly, 2 μM calcein-AM and 4 μM ethidium homodimer-1 were used to stain the constructs. First, the media was aspirated from around the construct, which was promptly washed with PBS. The construct was then submerged in the staining solution for 30 min, removed from the solution, and then tested using a Nikon Eclipse Ti2 (Nikon NIS-AR) confocal microscope. Cellular viability assays verified that there were no cytotoxic effects in the biomaterial when maintained in a 1.4 MHz magnetic field. No cytotoxic effects were consistent with previous studies for this bioink [43,44]. It is worth noting that some dead cells were observed for the initial cell seeding for both control and RF samples but were presumably washed away on subsequent days due to dead cells not adhering to the bioscaffold. 

### 2.4. Magnetic Field Instrumentation

An improved Helmholtz coil system was used to control the static and oscillating magnetic fields, as previously described [1]. A 6-channel DC power supply was used to control the 50 μT static magnetic field directionality in each of the two incubators. A triaxial magnetic field sensor provided PID controlled automatic feedback that allowed for real-time control of the three magnetic field axes within the incubators. The system can cancel out directional static magnetic fields in the other axes to approximately 20 nT. Therefore, the triaxial coil system cancels out ambient directional magnetic fields, including the geomagnetic field (20–60 µT), and is replaced by an applied 50 µT static magnetic field. A circuit printed Helmholtz coil was used for the oscillating magnetic field at 1.4 MHz. While the secondary RF coils existed in both incubators, only one RF configuration was powered in an incubator, labeled RF samples. The static magnetic field orientation was in the plane of the cells and the RF was parallel to the static magnetic field in the same plane. The RF magnetic field amplitude was set to 10 μT_RMS_. Two samples were studied simultaneously for each day: one control sample with only a static magnetic field at 50 µT and one sample with static and RF magnetic fields. 

### 2.5. DAPI-Phalloidin

Cellular constructs were incubated in either a 50 μT static field or 50 μT static field with 1.4 MHz RF for 0, 1, or 7 days. At such time points, constructs were washed with PBS and submerged in 3.7% formaldehyde for 15 min to fix the cells. The constructs were again washed with PBS and submerged in 0.1% Triton-X, a permeabilization buffer, for another 15 min. Following the manufacturer’s instructions, samples were again washed with PBS and submerged in DAPI-phalloidin (SigmaAldrich, St. Louis, MO, USA) for 1 h prior to imaging. Samples were then imaged on a Nikon Eclipse Ti2 confocal microscope.

### 2.6. Scanning Electron Microscopy

Cellular constructs were tested on days 0, 1, and 7 to measure the porosity and support materials of the samples as a function of time. First, cell samples were fixed with 3.7% formaldehyde for 1 h. All samples were dried in ethanol solutions, which increased in series by 10% every 10 min to reach 100% ethanol. Critical point drying (Denton Vacuum DCP-1, Moorestown, NJ, USA) was conducted to replace the ethanol with carbon dioxide. Samples were gold sputter coated (Denton Vacuum Desk IV) for 60 s. A second round of sputter coating occurred after rotating samples approximately 90° to ensure full coverage of the sample. Samples were observed with a JOEL JSM-6380LV (JEOL USA, Peabody, MA, USA) scanning electron microscope (SEM) with an acceleration voltage between 5 kV and 10 kV.

### 2.7. Image Processing 

Image processing was conducted with MATLAB routines to locate cells and to generate a coordinate system for calculating clustering rates. First, the images were converted to a binary image with a threshold value of 0.1. The images were then morphologically opened and closed using reconstruction to produce a noiseless image. Watershed segmentation was used to identify the cell centroids in the images. The centroids were assigned coordinates based on the image magnification. The coordinates of each centroid were stored and used for cell migration analysis. A detailed discussion of image processing can be found in the Supplementary Methods Section. 

### 2.8. Data Analysis

The cell centroid coordinates were used to measure the change in cell density over time. First, the cell distribution in the image geometric center was calculated. The cell distribution was then normalized relative to the number of cells in the image. Using a cumulative distribution histogram, a fourth-degree polynomial function was fit to the curve. This polynomial was used to determine the half-radius (R50) of the cell distributions in the images by solving for the polynomial when y = 0.5. Cell density was then determined by dividing half the number of cells identified by the area encompassed within the determined R50 for that image. Density was calculated in cells per micrometer squared. The cell densities were then compared for days 0, 1, and 7 to create a change in density over change in time. A detailed discussion of data analysis can be found in the Supplementary Methods Section. 

## 3. Results

### 3.1. Nuclei and Cytoskeleton Imaging

**Figure 2:**
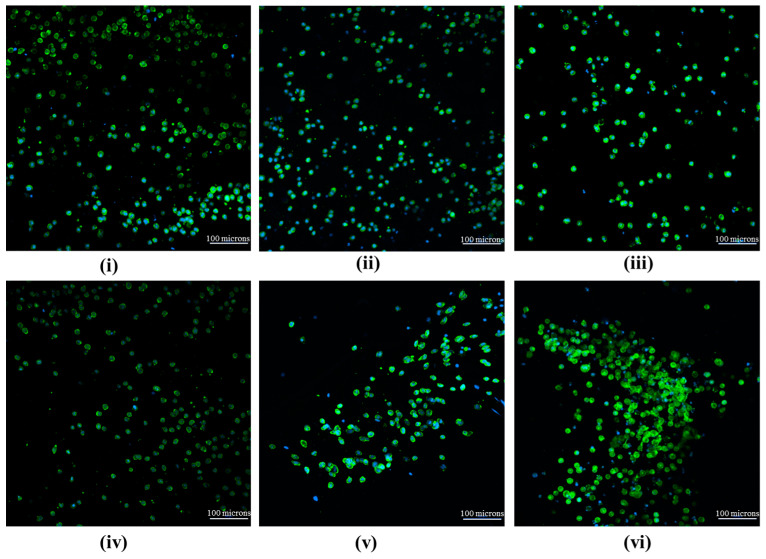
(**Top**) Nuclei and cytoskeleton staining demonstrating the distribution of cells (**i**) initially after construct synthesis, (**ii**) after 1 day at 50 μT, and (**iii**) after 7 days at 50 μT. (**Bottom**) Nuclei and cytoskeleton staining demonstrating the distribution of cells (**iv**) initially after construct synthesis, (**v**) after 1 day at 50 μT + RF, and (**vi**) after 7 days at 50 μT + RF. The scale bars are 100 µm.

DAPI and phalloidin staining were used to label the cells’ nuclei and cytoskeleton, respectively, as depicted in Figure 2. Day 0 samples showed a homogenous distribution of the cells throughout the construct, which was expected based on a similar distribution from the live/dead assay. Migration was observed based on characteristic elongation of the cells. The clustering for RF-maintained samples continued occurring over the course of the seven days. Multiple sections of each sampled construct showed that the clustering occurred throughout, but elongation ceased once the cytoskeletons of multiple cells began to overlap. The cell linkage was observed for the day 7 RF samples, whereas the control samples exhibited minimal clustering and migration. The RF sample had similar network-like fibrillar features in human umbilical vein endothelial cell clusters in day 7 samples with fibrinogen included in the same bioink [43,44]. 

### 3.2. Surface Topography and CDM Formation

**Figure 3:**
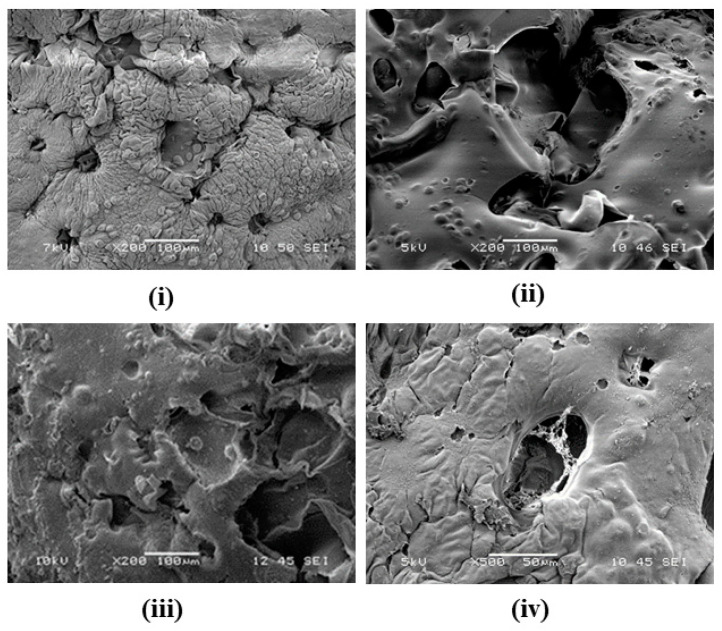
(**Top**) Sample SEM images taken at 200× of (**i**) cellular constructs on day 1 and (**ii**) cellular constructs on day 7. Both constructs shown were maintained in the static 50 μT magnetic field incubator. (**Bottom**) Sample SEM images taken at 200× for (**iii**) cellular constructs on day 1 and 500× for (**iv**) cellular constructs on day 7. Both constructs shown were maintained in the static 50 μT magnetic field with 1.4 MHz incubator. The key feature is the CDM web formed in the cavities in (**iv**) and absent in (**ii**).

SEM images of constructs maintained in a 50 μT static field demonstrated the basic thermos-reversible properties of gelatin as well as the structural support that cells provide, as seen in Figure 3 (top). Cellular constructs on day 1 showed some evidence of cells on the scaffold surface based on the nodular surface topology compared to acellular day 0 images (see Supplementary Material Section S2.3). While scaffold degradation did occur and open cell pores were created more homogenously, the degradation was not as drastic as that in the acellular samples. This is because cells attached to the binding sites of the gelatin and maintained the prolonged structural integrity of the construct. Day 7 samples yielded similar results, but with a more significant surface area due to open cell pores. 

SEM images of constructs maintained in a 50 μT static field with 1.4 MHz demonstrated an increase in porosity, as seen in Figure 2 (bottom). While the construct maintained its general shape, the irregular topography enabled a significant amount of sun-spotting for the images and caused damage to all samples. Day 1 cellular constructs showed increased porosity, specifically for closed cell pores. Day 7 cellular constructs showed mesh-like networks commonly overlaying pores less than 40 μm. These mesh-like networks are indicative of fibers being deposited by the cells and can be considered part of the CDM. For studies with long durations, the addition of the CDM is expected to begin to cause gelatin to dissipate more readily, which is an indicator of cellular clustering.

### 3.3. Change in Cell Density

Clustering was determined for cells in both magnetic field environments for initial seeding, after 1 day of treatment, and after 7 days of treatment, Table 1. The cells maintained in RF had an increased rate of 6.6 × 10^−4^ cells·μm^−2^. From day 1 to day 7, the RF sample slowed clustering with a rate of 1.8 × 10^−4^ cells·μm^−2^ per day. This suggests that RF continues to increase clustering from the first day to the seventh day but does so at a slower rate over time. The control cells showed minimal clustering, with an average density of 6.2 ± 1.5 × 10^−4^ cells/µm^2^. 

## 4. Discussion

This work presents a QBC framework for the use of magnetic fields to study cell migration in 3D biological constructs. Related reports have shown magnetic field effects on cellular migration rates [45,46,47]. Our results can be interpreted as manifestations of the RPM and ROS partitioning that impact oxidative signaling channels by quantum processes at the point of origin. We have previously shown that this magnetic field configuration, 1.4 MHz parallel to 50 µT static magnetic fields, increased cellular respiration by the RPM and ROS oxidative signaling. Increased cellular respiration results in elevated ATP synthesis, which promotes cytoskeletal activity. Cytoskeletal activity is a prerequisite for cell migration, where collagen secretion and cellular adhesion are impacted. For this reason, it can be concluded that the addition of RF increases cellular mobility and the formation of the CDM. Therefore, increased cellular migration rates are attributed to an oxidative signaling adaptive response [30] facilitated by ROS-mediated spin resonance, affecting mitochondrial bioenergetics and cytoskeletal movement.

A full understanding of cellular migration in a 3D hydrogel is a complex process and is dependent on many factors [48]. Moreover, fibroblast proliferation and migration rates differ between 2D plates and 3D constructs. Fibroblast proliferation is enhanced on 2D growth plates, whereas fibroblast quiescence enhances migration in floating 3D constructs [49,50]. Matrix characteristics have been shown to impact cell–matrix interactions that affect both cell proliferation and migration [51]. The results presented here show cell elongation in RF samples that support enhanced migration and the accelerated formation of the CDM in cell clusters. Therefore, migration is remotely enhanced by RF intervention, where changes in migration rates are best observed at RP magnetic resonance. The cells coalesce towards each other rather than a preferred directionality of the magnetic field. This observation is indicative of magnetic resonance impacting the RPM and ROS signaling, which leads to a magnetic mitohormesis response and not a mechanical mechanism. 

A novel method was developed that determines changes in cell density over time. This method allows for the distribution of the cells to be captured and uses this distribution to describe clustering rates, i.e., cell density changes over time to determine the clustering rate. This gives a time-dependent metric for the total migration of cells within a sample, allowing one to transition from visual inspection of cell images to a mathematical model that can describe cellular migration. Notably, this approach allows for a more quantitative analysis of cell migration time-dependence and provides a route for direct numerical comparison between samples. Without this novel analysis, any data depicted with regards to cell clustering would be necessarily more qualitative. From our data analysis, we were able to determine the clustering rate over time for the RF-treated and the control samples. Through a direct comparison of these rates, we calculated that the RF-treated tissue clustered at a rate ~5 times faster than the control tissue during the first day of treatment and continued to increase clustering over seven days of treatment. This suggests that RF dramatically increases the migration rate of cells, allowing them to not only cluster much more, but to do so faster, reaffirming our hypothesis that parallel RF magnetic fields increase cell performance. 

SEM images provided topological information of the scaffold and verified that the degradation of acellular scaffolds occurred faster in RF environments. The development of fibrous networks and the CDM was visualized after 7 days in cellular samples maintained in RF. This bioink composition has only previously observed fibrous network formation after 7 days without a static field when fibrinogen was included [43,44]. The increased rate of CDM formation can potentially facilitate the acceleration of tissue engineering constructs for personalized medicine. 

Future work to support our ROS QB hypothesis would involve real-time ROS cell imaging to gain more insight into the spatial–temporal evolution of oxidative signaling. One real-time method is the combination of optical-detected magnetic resonance and microscopy (ODMRM), similar to methods used for nitrogen-vacancy centers in diamonds [52]. This would give us a deeper understanding of the RPM’s and ROS impact on biological function. Better ROS-specific measurements would lend information as to the population changes due to altered spin dynamics. There are many modular options for ODMRM technology, including hyperspectral, multispectral, and label-free ROS imaging [53]. Notably, advanced label-free imaging techniques offer real-time measurements of FAD and NAD(P)H, which are proxies for indirect ROS measurements and bioenergetics [54]. Moreover, live cell imaging with either endogenous fluorophores [9] or exogenous sensors [55] is poised for ROS QB studies. Other ROS sensing methods could also be effective for this kind of approach [56,57,58], as well as temporal multiplexed imaging [59]. A particular direction of interest is the integration of electric dipole interactions [60], voltages [37], optical imaging [61], and magnetic fields [9]. The methods proposed can be used to selectively monitor the spatial–temporal domains within the QBC in real-time live cells. 

In a broader context, QBC timing can be understood within the experimental design and observed results. Control cells under static magnetic field conditions undergo metabolism in synchronization across the spatial and temporal domains. By changing the timing at the RPM level and the point of ROS generation (nano- to microseconds), ROS partitioning disrupts the QBC timing in an asynchronous fashion that can lead to either a speedup or slowdown of cellular function. The redistribution of ROS products by flavoproteins leads to oxidative signaling that readjusts mitochondria bioenergetics between glycolysis and respiration. Here, we used a magnetic field configuration that increased cellular respiration, which elevated ATP levels and increased cytoskeleton activity to accelerate cellular migration. Fundamentally, changes in singlet-triplet mixing at RP timing have significant impacts on the QBC and, ultimately, on cellular function across the spatial–temporal domains. 

Physiological applications for ROS and QB exist in not only tissue engineering but also neurodegeneration and aging models [62,63]. Because of the parallel RF oscillating magnetic field’s ability to modulate ROS production, there is an avenue for treating certain diseases such as Alzheimer’s Disease (AD). Amyloid β plaques are a well-known characteristic of AD, but high levels of ROS accompany disease and are thought to precede these plaques [64,65]. Superoxide is the primary source of oxidative stress in the brain and leads to downstream damage in nervous system tissues. A possible way to alleviate oxidative stress in the brains of AD patients lies in the methods given in this paper. We have shown that RF has notable and dramatic effects on cell performance and predict that we can use these effects to treat diseased neurons. Biomedical applications are waiting to be tapped into; for example, the method outlined here can be used to study the hallmarks of aging [66], stem-cell fate [67,68], and personalized medicine [69]. One could analyze the change in ROS production using specific sensors, allowing for accurate measurements of efficacy, cellular outcomes, and timing. In clinical settings, applications are being developed for diabetes [15], wound healing [70], regenerative medicine [11], and inflammation [4] that provide remote magnetic field intervention. Magnetic field intervention can provide specific and timely results with limited adverse side effects, offering an auxiliary method in addition to conventional treatment avenues.

### Study Limitations

There are certain limitations of our study that need to be highlighted. While the hypothesis and experimental procedures are robust, more experiments are needed to authenticate the statistical nature of the results and to determine the optimal impact of magnetic field intervention. Moreover, the computer vision algorithms have some preprocessing and thresholds that are adjusted to improve consistency across images. For example, down-sampling pixel density was needed to reduce noise and conserve signal intensity. An optimal pixel density and intensity seemed to correspond to the size of cells and features within the images. Lastly, due to the constraints of the RPM operation in living systems, other mechanisms of interaction cannot be ruled out. However, the hypothesis was to use experimental parameters to affect the RPM, ROS, bioenergetics, and thus cellular migration. More detailed experiments would be needed to connect bioenergetics and cytoskeleton activity, as well as any connection with calcium signaling.

## 5. Conclusions

With more evidence of the RPM in cellular ROS production and magnetic mitohormesis adaptive responses, we propose a novel framework that introduces a type of QBC. The QBC is a general framework and experimental approach for a better understanding of cellular timing across spatial–temporal domains. The QBC operates throughout selected spatial–temporal domains within normal cellular function in synchronous modes. Magnetic resonances alter the singlet–triplet mixing at the point of ROS generation, thus altering the cellular timing of the QBC in an asynchronous fashion. The cells respond to the QBC by adapting to the activated oxidative signaling channels that percolate through the cellular spatial–temporal domains. In this manner, the QBC can potentially impact cellular timing using a novel and fundamental mechanism, either by slowing down or speeding up cell time, affecting cell migration rates. This work represents a manifestation of ROS QB and supports evidence of a novel QBC operating as a control system that originates at the quantum level.

## Figures and Tables

**Table 1:** Calculated cell densities between control and RF experiments.

Sample	R50 (µm)	Number of Cells	Density (Cell/µm^2^)
Control Day 0	259	263	6.2 × 10^−4^
Control Day 1	251	300	7.6 × 10^−4^
Control Day 7	233	159	4.7 × 10^−4^
RF Day 0	218	177	5.9 × 10^−4^
RF Day 1	158	195	12.5 × 10^−4^
RF Day 7	161	385	23.5 × 10^−4^

## Data Availability

The datasets for this study can be found in the github repository at https://github.com/Cameron-McNamee/Cluster-Analyzer, accessed on 31 August 2022.

## Supplementary Materials

The following supporting information can be downloaded at: https://www.mdpi.com/article/10.3390/bioengineering11010009/s1.
