# Supplementary material for: Magnetic Field Intervention Enhances Cellular Migration Rates in Biological Scaffolds
Source: Bioengineering (Basel). 2023 Dec 22;11(1):9. doi: 10.3390/bioengineering11010009 (PMC10813414; doi:10.3390/bioengineering11010009)
Supplement: Supplementary file 1 [file bioengineering-11-00009-s001.zip › bioengineering-2740010-supplementary.pdf]

Supplementary Material

1 Supplementary Methods

1.1 Image Processing

The raw images were first edited to be 750-by-750 pixels (Figure S1A) with 120 pixels = 100 microns. This was done to ensure consistency across all images and to improve accuracy. These dimensions were found to be optimal for providing the highest image quality while allowing the algorithm to run with the highest speed. Higher pixel density images were attempted, and they revealed levels of noise that could not be removed without also removing substantial portions of signal. Lower pixel density images lead to inaccuracies with the image processing, causing the algorithm to misidentify cells because of the high loss in the noise removal process.

Images were then converted to grayscale (Figure S1B). This was done as an intermediate step towards binarizing the images. The grayscale images were then binarized using a luminance threshold of 0.1 (Figure S1C). Otsu's method was attempted, along with an adaptive threshold. Otsu's method was not detailed enough and lead high amounts of loss for fainter cells. This is likely due to the low difference between the foreground and background in certain areas. An adaptive threshold picked up extreme amounts of noise, leading to entire sections of images coming up as signal when there were clearly no cells present.

The binary image was morphologically eroded by a disk of radius five. The eroded image was then used as a marker and the original binary image as a mask for morphological reconstruction. This process effectively completed morphological opening. Opening was attempted directly, but it removed too much signal and yielded an image that greatly deviated from the original. Reconstruction has the added advantage of producing an image that is more like the original, leading to higher accuracy and lower loss.

The opened image was then morphologically dilated using a disk of radius five. The compliment of the dilated image was used as a marker and the compliment of the opened image as a mask for morphological reconstruction. The compliment of the image produced was then taken. Similarly, this process was effectively morphological closing. For the same reasons outlined above, closing was not used directly (Figure S1D).

After noise removal, the image was segmented using watershed segmentation (Figure S1E). The reason for segmenting was to produce a more accurate dataset that identified each cell. This process was attempted without segmentation, and it was unable to identify closely linked cells that were obviously not individual cells. With the addition of segmentation, the datasets were much more representative of the raw images. The watershed segmentation was done by first converting the modified image to a distance transform. The compliment of this transform was used to create the watershed. The pixels that were background in the binary image were set to zero in the watershed. This produced a labeled image that could then be used for identifying the individual cells.

The centroids of each label were found and stored as the locations of the cells (Figure S1F). This completes the image processing.

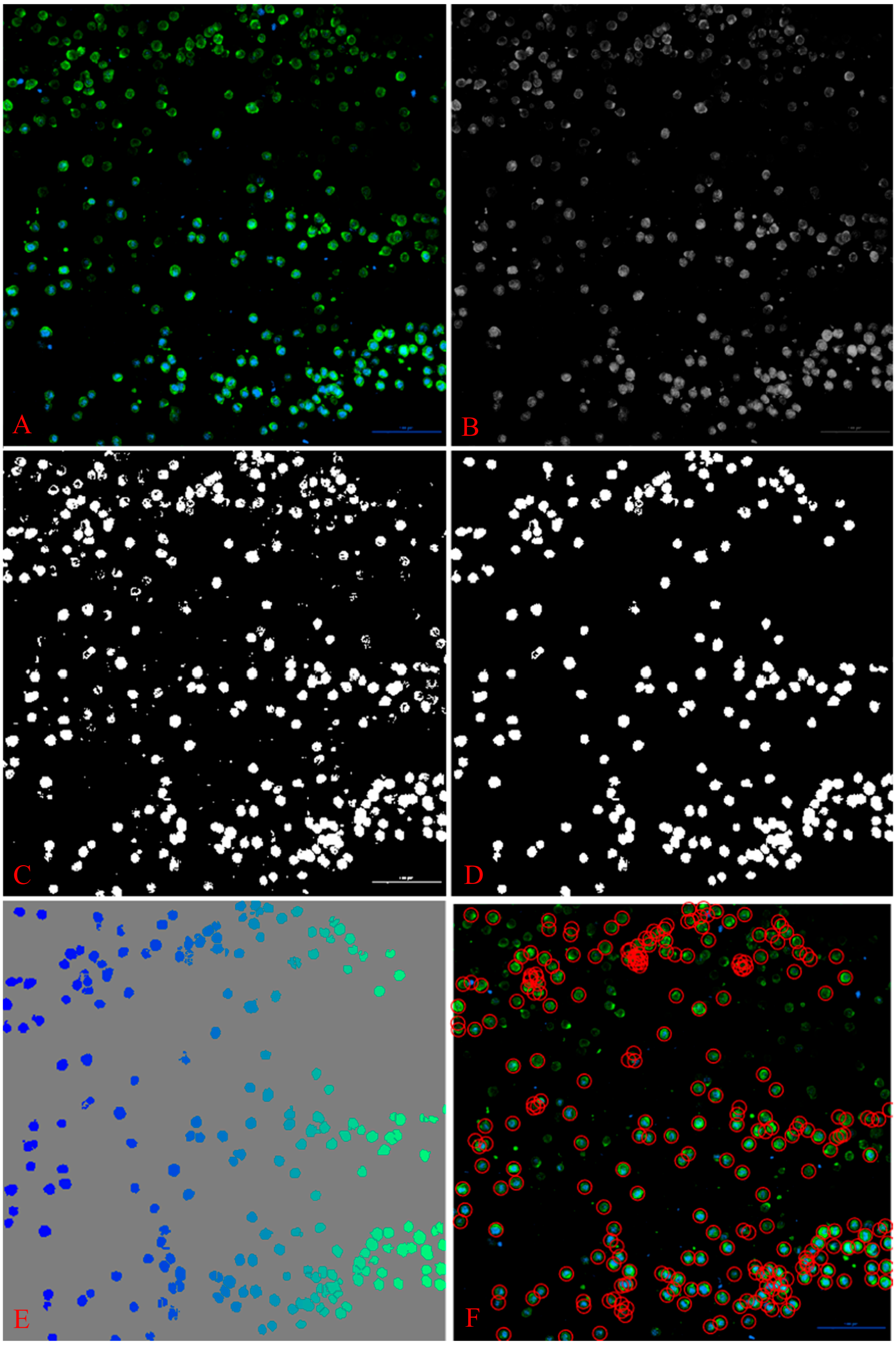

Supplementary Figure S1. (From left to right, top to bottom) The image used here is the control sample upon initial seeding. **(A)** The raw image that has been converted to 750-by-750 pixels **(B)** The image once it has been converted to grayscale **(C)** The grayscale image after being converted to a binary image **(D)** The binary image after undergoing the noise removal process **(E)** The labeled map

produced after segmenting the noise-removed binary image using watershed segmentation **(F)** The cells identified by the centroids of the labels superimposed on the original image. Here, one can see the robustness of the algorithm. Picking up faint cells that may be identified with the naked eye is not plausible without also picking up on high amounts of background noise.

1.2 Data Analysis

Once the cells were identified, the distribution was able to be determined. This was done by first identifying the geometric center of the cells (Figure S2i),

$$x_{mean} = \frac{\sum_{c \in C} c_x}{|C|}$$

$$y_{mean} = \frac{\sum_{c \in C} c_y}{|C|}$$

where C is the set of all cells, c_x and c_y are the x- and y-coordinates of a given cell c. With this as the reference point, the distance of each cell to this center was then calculated.

$$d_c = \sqrt{(x_{mean} - c_x)^2 + (y_{mean} - c_y)^2};\ \forall c \in C$$

With these distances, a normalized histogram was produced. This showed the relative number of cells within a given radius (Figure S2ii). Using this graph, a degree four polynomial was fitted to the curve (Figure S2iii). This x-values of the polynomial when equal to 0.5 were then found.

$$p_4x^4 + p_3x^3 + p_2x^2 + p_1x + p_0 = 0.5$$

Imaginary and non-positive roots were excluded. This was called the half-radius or R50. This radius was the minimum radius required to produce a circle, centered about the geometric center, to include half the total number of cells in the image (Figure S2i). The reasons for calculating the radius containing half the total number of cells, rather than some other amount, were: 1) having a radius dependent on a smaller proportion of the cells did not accurately depict the entire population of the tissue. This was because a smaller radius would be highly dependent on the cells surrounding the immediate area about the geometric center. This gives a poor metric of the entire picture. 2) A radius including a higher proportion of the cells would be highly dependent on outliers. This would cause images with cells around the border to have disproportionately high R50's, which is not an accurate measure of clustering.

With the R50 calculated, cell density was determined.

$$\rho = \frac{|C|}{2\pi(r_{50})^2}$$

This gives a density in cells per micrometer^2. The reason for having 2 in the denominator is to divide the number of cells in half. This way, we consider half the number of cells over the area needed to cover the same number of cells. This does not make a difference when comparing the rates between one another, but nevertheless gives more sensible values that correspond with what is being done in

theory. With this calculated density, we then compared the different images over time to determine the clustering rate.

$$\frac{\rho_a - \rho_b}{a - b} = \frac{\Delta\rho}{\Delta t}$$

With a direct comparison of these calculated rates, we were able to show the increased clustering rates that supported our hypothesis.

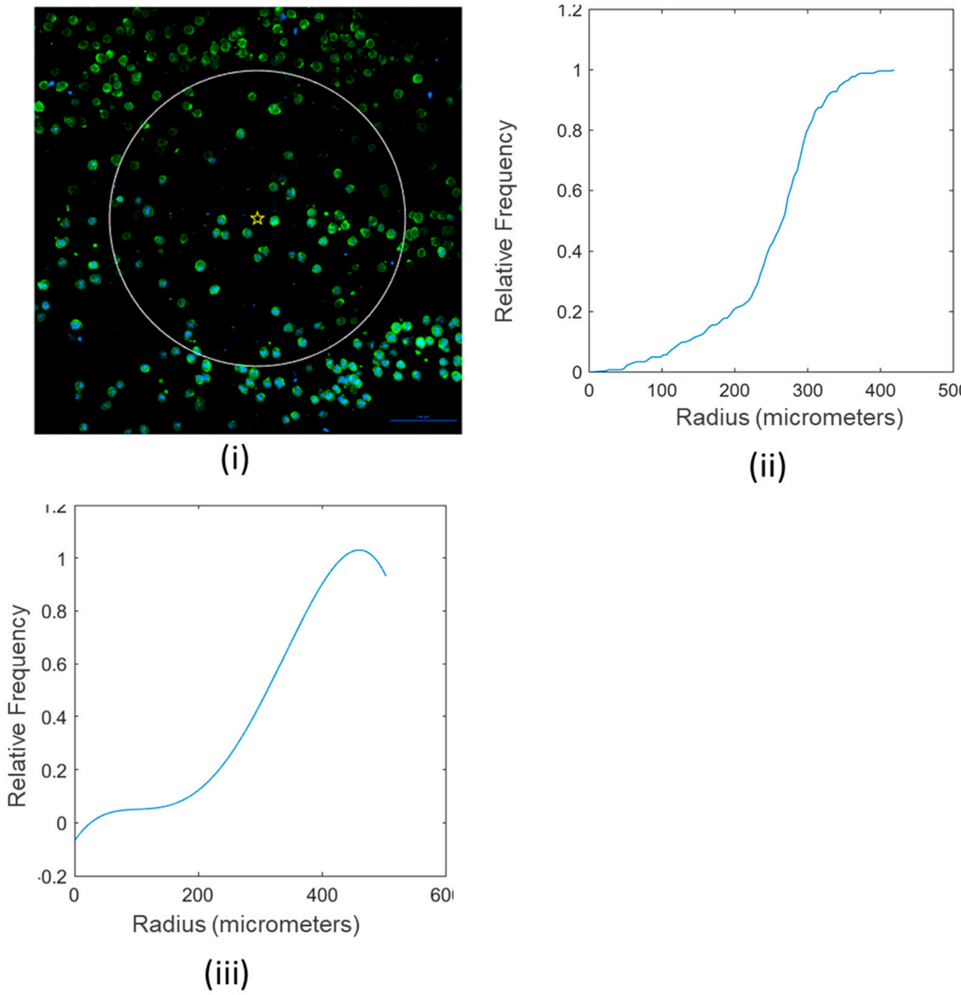

Supplementary Figure S2. The image used here is the same as above, control sample upon initial seeding. **(i)** The geometric center drawn as the gold pentagram near the center and the circle described by the calculated R50 for this image. **(ii)** The graph of the distribution about the geometric center. For a given radius (x), the relative frequency is the corresponding value (y). Here, relative means the number of cells within a radius divided by the total number of cells. This means when y=0.5, the radius encompasses half the cells. **(iii)** A degree four polynomial fitted to the curve described in **(ii)**.

2.1 Cellular Viability

Live/Dead assay was conducted at day 0 and day 1, as seen in Figure S3, to verify that cells were able to survive in the magnetic field. No cytotoxic effects were noted for this bioink composition in previous studies [6,23]. Cytotoxicity assay using calcein-AM (green) and ethidium homodimer-1 (red) on (i) day 0, (ii) 1 day in the static field, and (iii) 1 day in the static field + RF. Day 0 images demonstrated the homogenous distribution of cells throughout the constructs. Additionally, this was the only sample type that contained fragments of dead cells. These were most likely damaged during the printing process due to shear stress on the cells. Day 1 constructs maintained in a 50 µT static field yielded similar results to the day 0 constructs in terms of cell density and homogeneity. It is believed that due to a lack of attachment, any dead cells remaining after 1 day would be easily washed away during the staining process as the scaffold is porous and there would be no cellular attachments. Day 1 constructs maintained in a 50 µT static field with 1.4 MHz showed no cytotoxic activity due to the addition of RF to the cells or biomaterial. For this reason, the bioink was deemed compatible with the experimental protocol and able to be used for the remainder of the study.

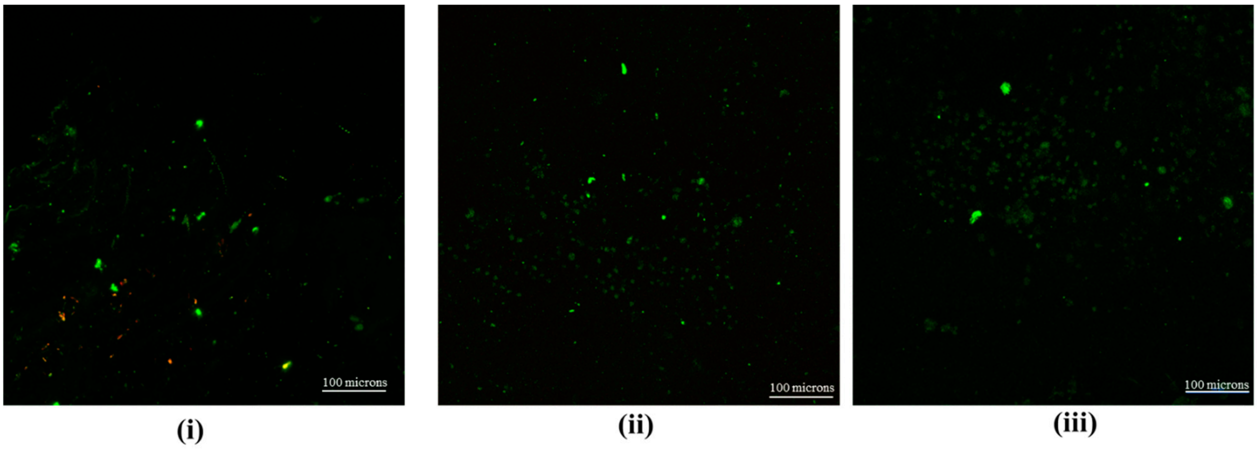

Supplementary Figure S3. Cytotoxicity assay using calcein-AM (green) and ethidium homodimer-1 (red) on (i) day 0, (ii) 1 day in the static field, and (iii) 1 day in the static field + RF.

2.3 Initial SEM images of Cellular and Acellular Constructs.

Cellular and acellular SEM samples were collected immediately after their synthesis, Figure S4. The acellular images demonstrated minimal open cell pores and no closed cell pores. Cellular constructs showed both minimal open cell pores and some evidence of open cell pores.

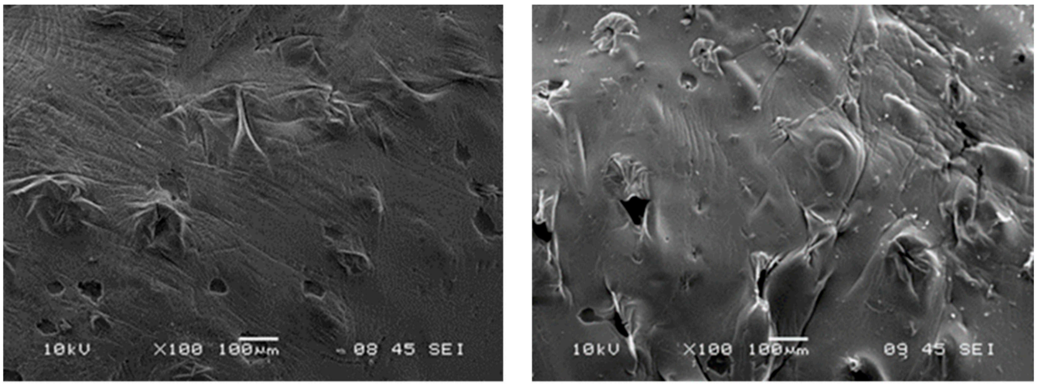

(i) (ii)

Supplementary Figure S4. Sample SEM images taken (i) day 0 acellular constructs and (ii) day 0 cellular constructs.
